# Severe Perinuclear Antineutrophil Cytoplasmic Antibody (p-ANCA)-Positive Granulomatosis With Polyangiitis

**DOI:** 10.7759/cureus.88082

**Published:** 2025-07-16

**Authors:** Sara Amer, Gizem Keles, Ami Shah, Nicole Melendez

**Affiliations:** 1 Internal Medicine, Lake Erie College of Osteopathic Medicine, Tampa, USA; 2 Rheumatology, St. Joseph's Hospital, Tampa, USA

**Keywords:** anca associated vasculitis, granulomatosis with polyangiitis (gpa), massive hemoptysis, pauci-immune crescentic glomerulonephritis, small vessel vasculitis

## Abstract

This case report describes a 50-year-old Caucasian male with a history of heroin use and hepatitis C who presented with upper respiratory tract, lower respiratory tract, and renal involvement, ultimately diagnosed with perinuclear antineutrophil cytoplasmic antibody (p-ANCA)-positive granulomatosis with polyangiitis (GPA). The patient initially presented to the emergency department with epigastric pain, leg pain, dyspnea, and hemoptysis, accompanied by recent ear infection, olfactory disturbances, and skin manifestations. Despite initial empiric treatment for suspected pneumonia, his condition rapidly deteriorated. Further investigation revealed elevated inflammatory markers, positive p-ANCA, and characteristic imaging findings including pulmonary nodules and cavitary lesions. Renal involvement was evident through hematuria and proteinuria, while ear, nose, and throat (ENT) examination showed chronic sinusitis and nasal crusting. Renal biopsy revealed pauci-immune necrotizing crescentic glomerulonephritis with evidence of granulomatous inflammation, serologic testing revealed p-ANCA positivity, and negative renal immunofluorescence microscopy results. Treatment was initiated with high-dose glucocorticoids and rituximab, considering the patient’s hepatitis C history. This case emphasizes the importance of considering GPA in patients with multi-system involvement, even with p-ANCA positivity, and highlights the complexities of managing patients with significant comorbidities.

## Introduction

Vasculitis refers to the inflammation of blood vessels, which can result in vessel wall damage, narrowing, obstruction, or rupture [1]. It can affect vessels of any size and may involve a wide range of organ systems. Classification of vasculitides is based on the 2012 Chapel Hill Consensus Nomenclature Conference [2]. Important to note, the classification is not rigid, as a typical small-vessel vasculitis can present in a medium-vessel and vice versa. Though based on the classification system, small-vessel vasculitides can be broken down further into antineutrophil cytoplasmic antibody (ANCA)-associated and non-ANCA-associated.

ANCA-associated vasculitides include granulomatosis with polyangiitis (GPA), microscopic polyangiitis (MPA), and eosinophilic granulomatosis with polyangiitis (EGPA). GPA commonly involves the upper and lower respiratory tracts as well as the kidneys [3-5]. MPA primarily affects the lungs and kidneys and is characterized by the absence of granulomas [4]. EGPA is distinguished by asthma, peripheral eosinophilia, and granulomatous inflammation [3-5]. Each of these conditions is typically associated with a specific type of ANCA, either c-ANCA (proteinase-3 (PR3) antibody) pattern or perinuclear antineutrophil cytoplasmic antibody (p-ANCA) (myeloperoxidase (MPO)) [3-5]. In contrast, small-vessel vasculitides, such as cutaneous small-vessel vasculitis (CSVV), IgA vasculitis (Henoch-Schönlein purpura), and cryoglobulinemic vasculitis, are not linked to ANCA antibodies [3-5].

From there, medium-vessel vasculitides include Kawasaki disease and polyarteritis nodosa (PAN) [3-5]. Kawasaki presents in children under the age of five typically with the classic conjunctivitis, rash, adenopathy, strawberry tongue, hand/foot changes, and a fever for five or more days [3-5]. PAN presents in adults 45-65 with a male predilection and involves neurological dysfunctions, renal impairment, rashes, and nodules. It spares the lungs and is associated with positive hepatitis B (HBV) or hepatitis C (HCV) studies, negative ANCA, and transmural vasculitis on muscle biopsy [3-5].

Finally, the large-vessel vasculitides include giant cell arteritis and Takayasu arteritis. Giant cell arteritis most commonly affects women over the age of 50, presenting with new-onset headache, a tender temporal artery, jaw claudication, and is typically associated with polymyalgia rheumatica [3-5]. Takayasu, on the other hand, most commonly affects Asian women under the age of 40. It is known as the pulseless disease due to disparity in blood pressures between arms and can present with a bruit over the subclavian artery or abdominal aorta [3-5].

Diagnosing ANCA-associated vasculitis and determining its specific subtype is challenging due to overlapping symptoms and laboratory findings. Furthermore, GPA does not have specific diagnostic criteria; therefore, this highlights the importance of ANCA titers, biopsy for vasculitis, and the overall clinical manifestations of systemic disease [6]. GPA is typically associated with a c-ANCA pattern, while p-ANCA GPA is seen in less than 20% of cases [1,6]. Additionally, approximately 10% of GPA cases are ANCA-negative, potentially leading to many undiagnosed patients [1]. Here, we present a rare case of p-ANCA-associated GPA presenting initially in an emergency department setting.

## Case presentation

A 50-year-old Caucasian male with a past medical history of HCV and previous intravenous heroin use presented to the emergency department with complaints of epigastric pain, right leg pain, progressive shortness of breath, and hemoptysis. He reported that his symptoms developed gradually over several days. Further review of systems revealed that he had recently experienced an ear infection, decreased sense of smell, a petechial rash on his left foot, and numbness and tingling in both lower extremities.

On initial examination, the patient was hemodynamically stable. Respiratory rate was mildly elevated, but oxygen saturation remained within normal limits on room air. Physical examination revealed clear lung fields on auscultation, normal heart sounds without murmurs, rubs, or gallops, and a soft, non-tender abdomen without organomegaly or palpable masses. A petechial rash was noted on the left foot. Neurologic exam demonstrated reduced sensation to light touch in the lower extremities. 

**Figure 1:**
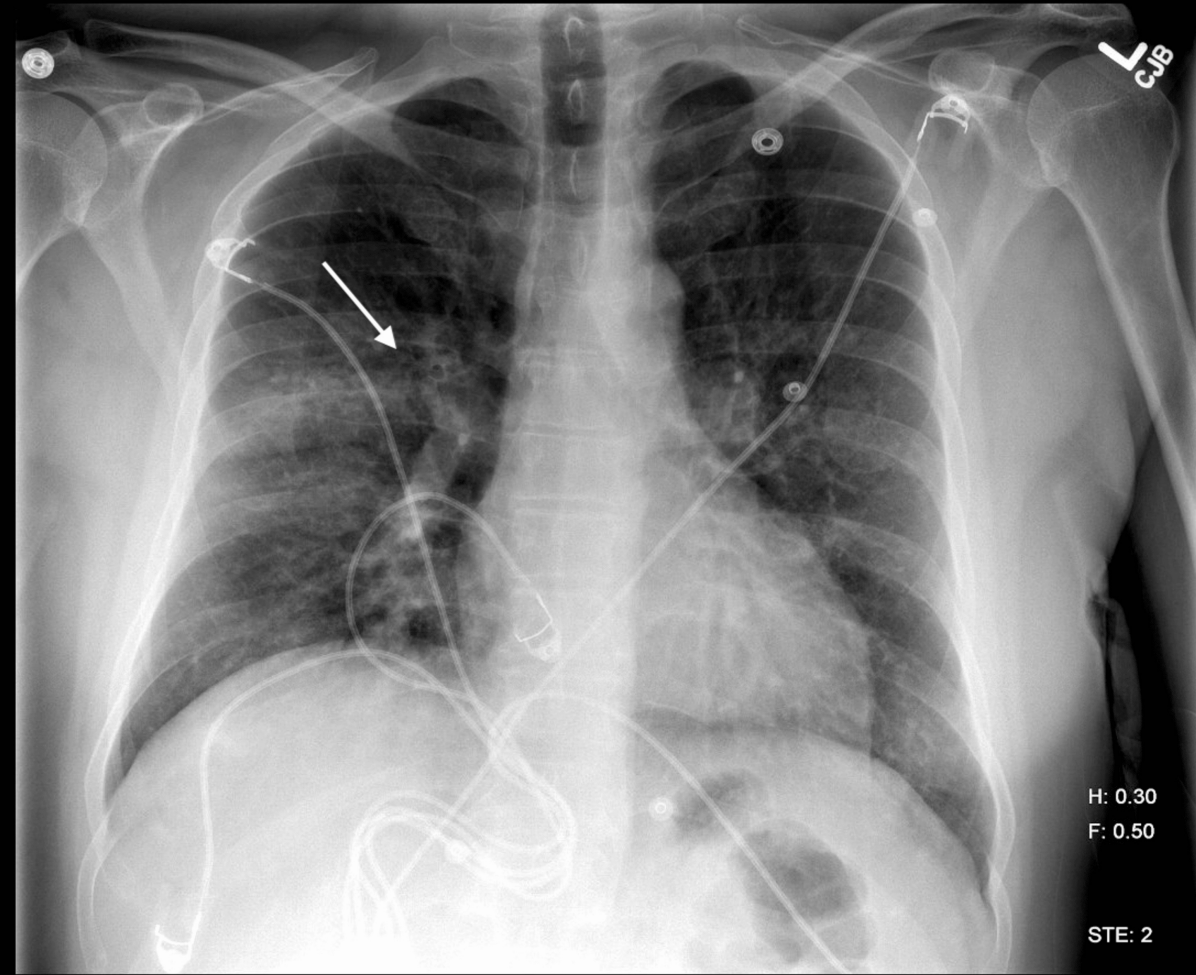
Chest radiograph demonstrates a right perihilar lower lobe infiltrate and ill-defined opacity in the left lower lobe, indicating possible infectious etiology.

A chest X-ray, seen in Figure 1, obtained at presentation revealed a right perihilar lower lobe infiltrate with a questionable ill-defined opacity in the left lower lobe, initially suspected to be infectious in origin. Laboratory evaluation at that time showed stable renal function, as seen in Table 1. The patient was empirically started on intravenous vancomycin, ceftriaxone, and ampicillin for presumed pneumonia.

**Table 1:** The patient’s laboratory values over the course of hospitalization demonstrated a rapid clinical decline characterized by progressive and severe multi-organ impairment ABG, arterial blood gas; BMP, basic metabolic panel; BUN, blood urea nitrogen; CBC, complete blood count; CRP, C-reactive protein; HCO₃, bicarbonate; HCT, hematocrit; HGB, hemoglobin; MCH, mean corpuscular hemoglobin; MCHC, mean corpuscular hemoglobin concentration; MCV, mean corpuscular volume; PaCO₂, partial pressure of carbon dioxide in arterial blood; PaO₂, partial pressure of oxygen in arterial blood; pH, potential of hydrogen; PLT, platelets; RBC, red blood cells; RDW, red cell distribution width; WBC, white blood cells

Category	Parameter	May 8, 2024	May 18, 2024	June 4, 2024	Reference Range
CBC	WBC (×10³/µL)	8.4	H 27.8	7.1	4.5-11.0
	RBC (×10⁶/µL)	2.60	L 2.80	L 2.63	4.35-5.65
	HGB (g/dL)	7.8	L 8.4	L 9.0	13.5-17.5
	HCT (%)	23.3	L 24.6	L 26.9	41-53
	PLT (×10³/µL)	270	L 111	247	140-400
	MCV (fL)	80.6	88.0	H 102.4	80-100
	MCH (pg)	26.8	30.1	H 34.4	27-33
	MCHC (g/dL)	33.3	34.1	33.6	32-36
	RDW (%)	12.9	H 15.7	H 16.5	<14.5
BMP	Sodium (mmol/L)	137	L 134	136	135-145
	Potassium (mmol/L)	2.7	3.9	L 3.3	3.5-5.1
	Chloride (mmol/L)	106	102	105	98-107
	Carbon Dioxide (mmol/L)	22	23	22	23-30
	Glucose (mg/dL)	91	H 125	83	Fasting: 70-99
	BUN (mg/dL)	80	H 57	H 103	7-25
	Creatinine (mg/dL)	4.760	H 2.100	H 1.920	Male: 0.7-1.3
	BUN/Creatinine Ratio	16.80	27.14	53.64	10:1-20:1
Urinalysis	Protein	3+	3+	4+	Negative
	Blood	3+	3+	3+	Negative
Inflammatory	CRP	9.00	6.00	12.00	Men Range: 0-3 mg/L Normal: <0.55 mg/L
ABG	pH	7.42	7.40	7.41	Arterial: 7.35-7.45
	PaCO_2_ (mmHg)	40.1	H 47.4	42	Arterial: 35-45
	PaO_2_ (mmHg)	73	L 67.8	94	Arterial: 80-100
	HCO_3_ (mmol/L)	28.4	H 29.3	26.6	Arterial: 22-28

Despite appropriate antibiotic coverage, the patient’s condition deteriorated rapidly. He developed acute renal failure, hypoxia, and respiratory distress. He required intubation, placement on extracorporeal membrane oxygenation (ECMO), and initiation of continuous renal replacement therapy (CRRT). Repeat imaging demonstrated worsening bilateral pulmonary infiltrates. Follow-up laboratory studies revealed rising blood urea nitrogen (BUN) and creatinine levels, as seen in Table 1, consistent with acute kidney injury.

Given the patient’s multisystem involvement and worsening clinical picture, several specialty teams were consulted. The nephrology service expressed concern for a possible ANCA-associated vasculitis based on the patient’s initial symptoms, imaging, and renal function trends. Hematology was consulted for evaluation of a prolonged activated partial thromboplastin time (aPTT), which did not correct on mixing studies. This raised suspicion for a lupus anticoagulant or an underlying hypercoagulable state, possibly linked to systemic inflammation. While awaiting serologic confirmation, the patient was started on therapeutic plasmapheresis due to concern for an underlying vasculitic process. 

**Figure 2:**
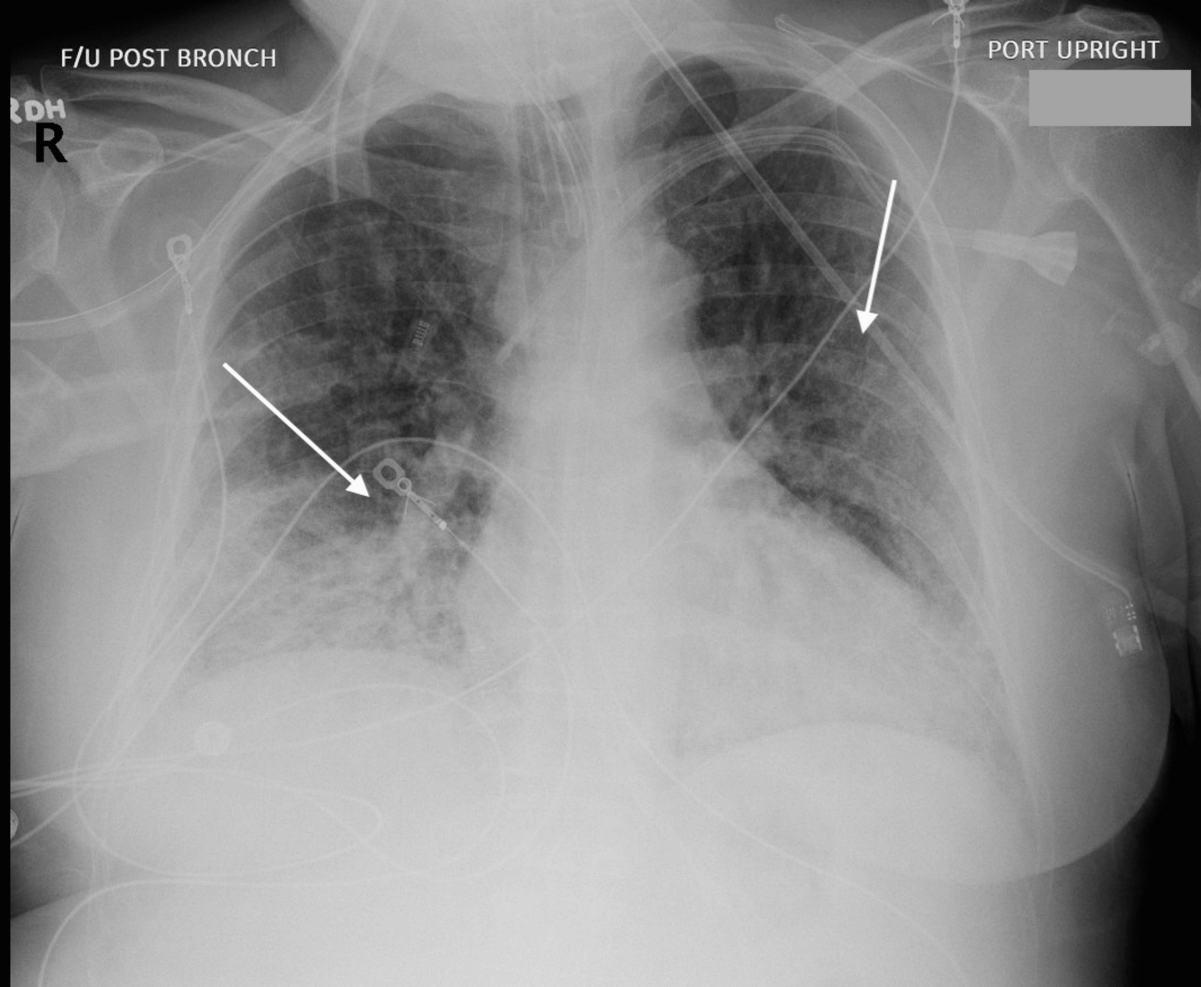
The chest radiograph following bronchoscopy demonstrates bilaterally increased interstitial markings, consistent with progression of respiratory distress.

**Figure 3:**
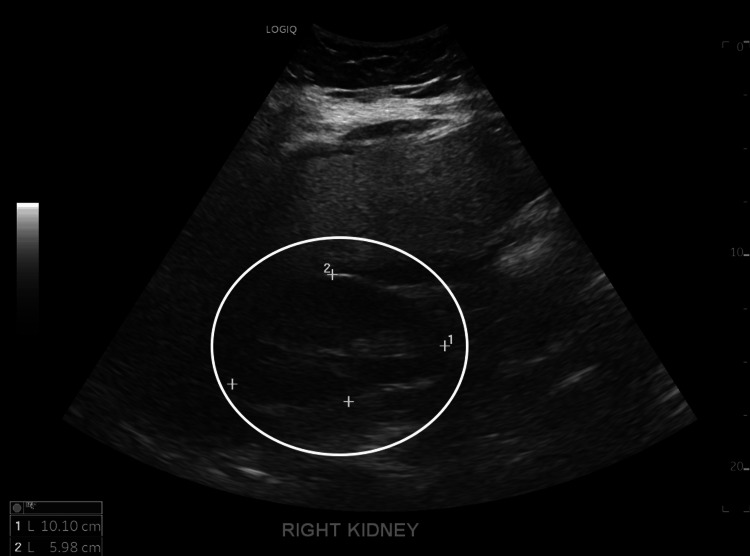
Right kidney ultrasound demonstrates increased echogenicity reflective of active glomerular injury and chronic fibrotic remodeling characteristic of antineutrophil cytoplasmic antibody (ANCA)-associated vasculitis.

**Figure 4:**
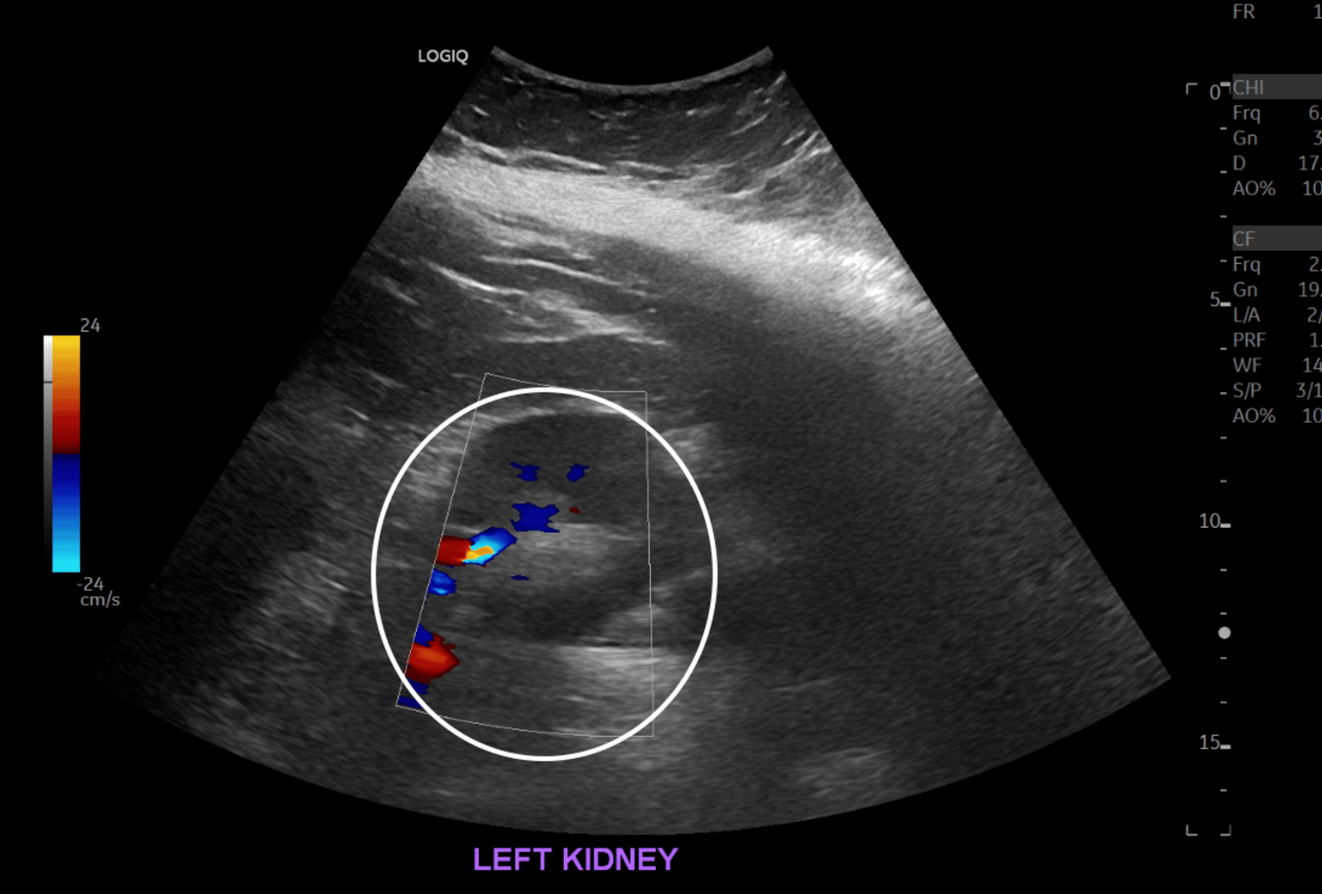
Left kidney ultrasound demonstrates increased echogenicity suggestive of ongoing glomerular inflammation and chronic fibrotic changes characteristic of antineutrophil cytoplasmic antibody (ANCA)-associated vasculitis.

The patient subsequently underwent bronchoscopy for evaluation of worsening hemoptysis. The procedure revealed active oozing of fresh blood in all bronchial subsegments, consistent with diffuse alveolar hemorrhage. Post-bronchoscopy imaging, seen in Figure 2, demonstrated significantly increased bilateral interstitial markings, in keeping with progressive respiratory failure. A renal ultrasound ordered by nephrology revealed increased echogenicity of the kidneys, as seen in Figure 3 and Figure 4, which is suggestive of active glomerular inflammation or underlying fibrotic damage consistent with ANCA-associated vasculitis [2]. 

The renal biopsy specimen contains 28 glomeruli, of which 20 are nonsclerotic and available for evaluation by light microscopy. Of these, a significant majority (20/28) demonstrate features of active glomerular injury, including fibrinoid necrosis and cellular to fibrocellular crescent formation, consistent with diffuse crescentic glomerulonephritis. No significant immune complex deposition is identified on immunofluorescence or electron microscopy, supporting a pauci-immune pattern. Scattered granulomatous inflammation is also noted within the interstitium, further supporting the diagnosis of a pauci-immune crescentic glomerulonephritis with granulomatous features. Renal immunofluorescence microscopy demonstrated no significant staining for IgG, IgA, IgM, C3, C1q, kappa, or lambda light chains, further confirming a pauci-immune process.

The patient was started on high-dose intravenous corticosteroids and rituximab for immunosuppression. Several days later, serologic studies returned positive for p-ANCA with MPO antibodies at a titer of 1:320. Additional testing for antinuclear antibody (ANA), anti-glomerular basement membrane (anti-GBM) antibodies, human immunodeficiency virus (HIV), and tuberculosis using the QuantiFERON Gold test was negative, as summarized in Table 2. These findings confirmed the diagnosis of p-ANCA-positive GPA, presenting with diffuse alveolar hemorrhage and rapidly progressive glomerulonephritis.

**Table 2:** Labs revealed p-ANCA titer positivity, while ANA, anti-GBM, HIV, and QuantiFERON tests are negative ANA, antinuclear antibody; GBM, glomerular basement membrane; HIV, human immunodeficiency virus; p-ANCA, perinuclear anti-neutrophil cytoplasmic antibody

Test	Current Result	Reference Interval
p-ANCA	1:320	Negative < 1:20, Positive ≥ 1:20
ANA	Negative	Negative
Anti-GBM	Negative	Negative
HIV	Negative	Negative
QuantiFERON-TB	Negative	Negative

The patient demonstrated a favorable response to the therapeutic regimen of plasmapheresis, rituximab, and corticosteroids, leading to marked clinical improvement and successful recovery, with a return to baseline functional status. He continues to follow up regularly with rheumatology and remains stable on maintenance rituximab therapy to prevent disease relapse. Ongoing laboratory monitoring is conducted to assess for potential complications and ensure sustained disease control.

## Discussion

ANCA-associated vasculitis includes various autoimmune conditions that involve necrotizing vasculitis and positive ANCA titers. The measured titers are directed against proteinase-3 (PR3), also known as c-ANCA, and MPO, also referred to as p-ANCA [2]. This case highlights the diagnostic complexity of ANCA-associated vasculitides, particularly when clinical features overlap. GPA, MPA, and EGPA are all forms of small-vessel vasculitis associated with ANCA positivity, yet they present with distinct pathological and clinical patterns. While MPA typically lacks granulomatous inflammation and predominantly involves the lungs and kidneys, and EGPA is defined by asthma and eosinophilia, GPA is uniquely characterized by necrotizing granulomatous inflammation involving the upper and lower respiratory tract as well as the renal system [3]. In this case, the patient's triad of involvement of ENT symptoms (sinusitis and nasal crusting), pulmonary manifestations (hemoptysis, nodules, and diffuse infiltrates), and renal impairment (hematuria, proteinuria, and elevated creatinine) strongly pointed toward GPA. Although GPA is predominantly associated with c-ANCA (anti-PR3) positivity, this case offers a valuable example of the less common p-ANCA (anti-MPO)-associated variant. p-ANCA positivity is observed in fewer than 20% of GPA cases, highlighting the rarity and clinical significance of this presentation [1,6]. This atypical serologic finding initially complicated the diagnostic process. However, the renal biopsy was definitive, revealing pauci-immune necrotizing crescentic glomerulonephritis with evidence of granulomatous inflammation, a histopathological hallmark of GPA. The absence of anti-GBM antibodies, negative ANA, and exclusion of other systemic conditions further narrowed the differential diagnosis. Taken together, the clinical presentation, serology, imaging findings, and confirmatory biopsy provided a comprehensive picture consistent with p-ANCA-positive GPA, distinguishing it from other forms of ANCA-associated vasculitis.

Early diagnosis of GPA or any vasculitis is crucial to prevent long-term complications. Severe cases of GPA were historically treated with glucocorticoids and cyclophosphamide. Treatment now includes the option of using rituximab instead of cyclophosphamide. This is in part due to the Rituximab for ANCA-Associated Vasculitis (RAVE) and Rituximab Versus Cyclophosphamide in ANCA-Associated Vasculitis (RITUXVAS) trials from 2010 [7,8]. The RAVE trial was a landmark double-blind, non-inferiority study comparing rituximab (375 mg/m² weekly × 4) to cyclophosphamide (2 mg/kg/day) for induction therapy in 197 patients with severe ANCA-associated vasculitis (AAV). At six months, rituximab demonstrated non-inferiority (64% vs. 53% remission, p < 0.001) and superiority in relapsing disease (67% vs. 42%, p = 0.01) [8]. Both groups had comparable safety profiles, though cyclophosphamide patients experienced more adverse events (33% vs. 22%, p = 0.01). Notably, rituximab led to higher PR3-ANCA negativity (51% vs. 17%, p < 0.001) and sustained remission at 18 months [8]. 

The RITUXVAS trial focused on 44 patients with severe renal AAV, randomized to rituximab (with two cyclophosphamide pulses) or cyclophosphamide followed by azathioprine [9]. At 12 months, sustained remission rates were equivalent (76% vs. 82%, p = 0.68), with no difference in severe adverse events (42% vs. 36%). Unlike RAVE, this open-label study included older patients (median age 68) and severe renal involvement, demonstrating rituximab’s efficacy in high-risk populations. However, the rituximab arm’s inclusion of cyclophosphamide pulses raised questions about its standalone role in renal disease [10]. However, both trials confirmed rituximab’s non-inferiority to cyclophosphamide, supporting its use as a first-line induction therapy, particularly in relapsing disease or cases where cyclophosphamide’s cumulative toxicity is a concern [11,12]. 

Plasmapheresis is also helpful as it helps remove ANCA from the blood in addition to other inflammatory mediators [13,14]. The patient responded well to plasmapheresis, rituximab, and corticosteroids, achieving full clinical recovery and return to baseline function, and remains stable on maintenance rituximab with regular rheumatology follow-up and ongoing laboratory monitoring.

## Conclusions

This case underscores the complexity of diagnosing and managing GPA, especially in patients presenting with multisystem involvement. The 50-year-old male initially presented with epigastric pain, dyspnea, and hemoptysis, along with ENT manifestations and renal dysfunction, reflecting the systemic nature of ANCA-associated vasculitis. Despite initial treatment with broad-spectrum antibiotics for presumed pneumonia, his condition rapidly progressed to acute respiratory distress syndrome (ARDS) and acute renal failure. Although GPA is most commonly associated with c-ANCA positivity, this patient exhibited a less typical p-ANCA pattern. Nevertheless, his constellation of upper and lower respiratory tract involvement, renal impairment, and histopathologic evidence of pauci-immune necrotizing crescentic glomerulonephritis with granulomatous inflammation strongly supported the diagnosis of GPA. The exclusion of other autoimmune conditions, including negative anti-GBM antibodies and lupus anticoagulant, further narrowed the differential. Ultimately, the integration of clinical presentation, serologic data, imaging studies, and renal biopsy findings was critical in distinguishing this atypical case of GPA from other forms of ANCA-associated vasculitis, such as MPA and EGPA. This case illustrates the diagnostic challenges posed by ANCA-associated vasculitides, particularly when serologic profiles deviate from classic patterns, and highlights the importance of a comprehensive, multidisciplinary approach.

Treatment with high-dose glucocorticoids, rituximab, and plasmapheresis aligned with evidence supporting rituximab’s efficacy in induction therapy, particularly given its comparable outcomes to cyclophosphamide in trials like RAVE and RITUXIVAS. Plasmapheresis played a critical role in mitigating inflammatory mediators during acute flares, though its utility remains debated outside severe renal or pulmonary hemorrhage. This case reinforces the need for early suspicion of vasculitis in patients with overlapping pulmonary, renal, and ENT symptoms. Timely immunosuppression and advanced supportive measures, such as ECMO, are vital to improving outcomes in life-threatening presentations. The patient’s history of HCV and heroin use, while not directly causative, highlights the importance of considering comorbidities that may complicate diagnosis and management.
